# Systematic Review of Trial Design and End Points in Lupus Nephritis

**DOI:** 10.1016/j.ekir.2026.106479

**Published:** 2026-03-10

**Authors:** Sophia Giang, Maria A. Dall'Era, Rachel B. Jones, Liz Lightstone, Jeanette Andersen, Elsa Martins, Jorge A. Ross Terres, Armando Turchetta, William F. Pendergraft, Ana Malvar

**Affiliations:** 1 Genentech, Inc., South San Francisco, California, USA; 2 Division of Pediatric Nephrology, Stanford Children’s Health, Palo Alto, California, USA; 3 Department of Nephrology, Cambridge University Hospitals NHS Foundation Trust, Cambridge, UK; 4 Department of Immunology and Inflammation, Imperial College London, London, UK; 5 Lupus Europe, Brussels, Belgium; 6 F. Hoffmann-La Roche Ltd, Basel, Switzerland; 7 Hoffmann-La Roche Ltd, Mississauga, Ontario, Canada; 8 Nephrology Unit, Hospital Fernández, Buenos Aires, Argentina

**Keywords:** autoimmune disease, clinical trial, inflammation, glomerulonephritis, lupus nephritis, study design

## Abstract

**Introduction:**

Advances in our understanding of immune dysregulation in lupus nephritis (LN) are driving a surge in the development of targeted pharmaceutical agents for a disease that continues to carry a high rate of morbidity and mortality. Although meaningful progress is being made in the management of LN, the substantial heterogeneity in critical elements of trial design and reporting that exists across contemporary LN trials hampers our ability to fairly compare the efficacy of new therapies.

**Methods:**

In this review, we comprehensively summarized and compared end points, various aspects of methodology (including glucocorticoid usage and eligibility criteria), actual trial population, and results reporting across studies published in the last 2 decades. We assessed 15 phase 2/3, interventional, randomized controlled trials (RCTs), of which 4 demonstrated the superiority of the investigational agent over standard therapy.

**Results:**

Emerging themes comprise the inclusion of participants with high median baseline kidney function (median of the average baseline estimated glomerular filtration rate (eGFR) was 95 ml/min per 1.73 m^2^), differences in definitions of active disease, high variability in kidney function, and glucocorticoid-based definitions of treatment response.

**Conclusion:**

Based on the findings of this review, we suggest a set of expert-posited guidelines to help in standardizing future studies in LN.

## Graphical abstract

**Figure:**
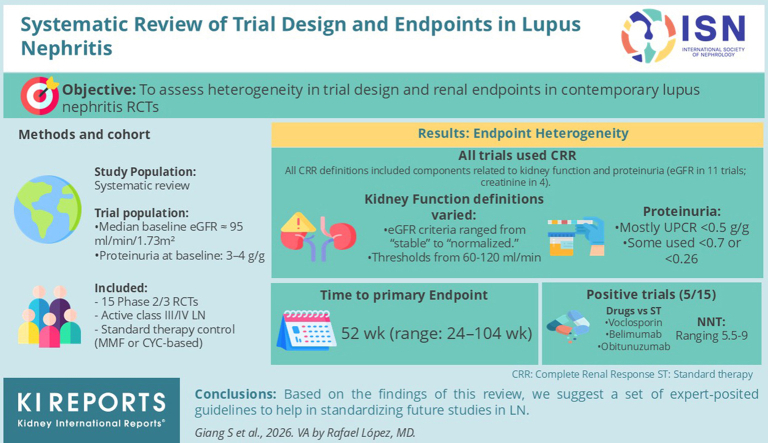

LN, the most severe organ manifestation of systemic lupus erythematosus (SLE), affects approximately 50% of patients with SLE.^1^ It carries a heavy burden, adding an approximately 2-fold risk for all-cause mortality compared with patients with SLE without LN and progressing to end-stage kidney disease (ESKD) within 10 years of diagnosis for 5% to 20% of patients.^2^^,^^3^ LN disproportionately affects socioeconomically disadvantaged populations, including racial and ethnic minorities, who face higher risks of poor health outcomes.^4^ Over the past 2 decades, research advances into LN pathogenesis have led to the development of novel therapeutics. Following years of limited pharmaceutical advancement, 3 novel agents (belimumab, voclosporin, and obinutuzumab) are now approved for active LN, with recent clinical guidelines conditionally recommending their addition to standard therapy (ST) upfront.^5^, ^6^, ^7^, ^8^ Although significant strides have been made, the treatment landscape for LN is still in the early steps toward precision medicine. Disease heterogeneity among individual patients presents an intrinsic challenge to optimizing outcomes, and the historic and ongoing heterogeneity of LN clinical trial design adds to this challenge. In this review, we document end points used in recent clinical trials, summarize the changes in recommended end points used in LN trials, critically evaluate elements of contemporary trial design and implementation to better contextualize the progress made in LN treatment, and provide recommendations to improve trial design and facilitate cross-trial comparison.

## Methods

This systematic literature review (SLR) was conducted in accordance with the Preferred Reporting Items for Systematic Reviews and Meta-Analyses 2020 guidelines.^9^ Searches were conducted in MEDLINE/PubMed, Cochrane Library, ClinicalTrials.gov, and EMBASE electronic databases from January 2013 to February 2025. Search terminology (Supplementary Table S1) filtered for records in English and included data from phase 2/3, multicenter RCTs involving LN treatments. The results were manually screened to identify trials of initial (formerly, “induction”) therapies that included patients with class III or IV LN, where the control treatment consisted of mycophenolate mofetil– or cyclophosphamide-based regimens. Included trials were primary studies (rather than *post hoc* analyses or trial extensions) with published results on treatment efficacy. Abstracts were included only if they contained sufficient details about methodology and efficacy results. Abstracts of studies later published as manuscripts were excluded.

Data pertaining to trial design, trial population, treatment regimens, definitions of end points used, and efficacy results from each trial were independently extracted by 3 authors using a standardized form (Supplementary Table S2). Summary statistics were completed in Stata BE 17.0.^10^ Two authors independently assessed the risk of bias (ROB) using the Cochrane Risk of Bias tool for RCTs.^11^ A third author adjudicated differences in ROB domain scoring. These assessments reflect ROB related to efficacy end points, which may not be the primary end point in some trials.

## Results

Overall, 162 unique trials were identified. Of these, 15 met the inclusion criteria following manual review for adherence to the predetermined eligibility criteria not integrated into the database search terminology (Supplementary Figure S1).^12^, ^13^, ^14^, ^15^, ^16^, ^17^, ^18^, ^19^, ^20^, ^21^, ^22^, ^23^, ^24^

### General Characteristics of Included Trials

A total of 15 trials were included: 7 phase 2 trials (including 1 abstract^25^); 7 phase 3 trials (including 1 abstract^26^), and 1 phase 2/3 trial. Sample sizes ranged from 43 to 448 participants (median: 226.5). Most (11/15, 71.4%) were international trials, spanning ≥ 2 continents. Fifty-three percent (8/15) of the trials had data published in or after 2020. The length of follow-up ranged from 24 to 104 weeks (Table 1).

**Table 1:** Basic study details for included trials

Investigational treatment (study)	Year published	Number of patients randomized	Intervention /noteworthy details of trial design	Primary end point	Total follow-up time (wks)	Met primary end point
OCR (BELONG)^13^	2013	381	Standard therapy (MMF or CYC for initial therapy, MMF, or AZA for subsequent therapy) + ocrelizumab 400 mg, ocrelizumab 1000 mg, or placebo	CRR, PRR, NRR at 48 wks	96	No
ABA^19^	2014	300	Standard therapy (MMF) + abatacept 10 mg/kg, high-dose abatacept 30 mg/kg, or placebo	Time to CRR	52	No
ABA (ACCESS)^16^	2014	134	Standard therapy (CYC for initial therapy, AZA for subsequent therapy) + abatacept or placebo / Withdrew treatment besides GC at 28 wks for responders	CRR at 24 wks	52	No
BIIB023 (ATLAS)^25^	2016	188	Standard therapy (MMF) + BIIB023 3 mg/kg, BIIB023 20 mg/kg, or placebo / Excluded patients with UPCR ≤ 0.5 mg/mg at 12 wks	CRR, PRR at 52 wks	52	No
TAC (TTT)^14^	2018	84	Standard therapy (MMF) vs. tacrolimus for initial therapy; MMF/AZA/CYC for subsequent therapy	SLEDAI-2K at 26 and 52 wks	52	No
ABA (ALLURE)^26^	2018	405	Standard therapy (MMF) + high-dose abatacept or placebo	CRR at 52 wks	156	No
VOC (AURA-LV)^12^^a^	2019	265	Standard therapy (MMF) + voclosporin 23.7 mg, voclosporin 39.5 mg, or placebo	CRR at 24 wks	48	Yes
BEL (BLISS-LN)^18^^a^	2020	448	Standard therapy (MMF or CYC for initial therapy, MMF, or AZA for subsequent therapy) + belimumab 10 mg/kg or placebo	PERR at 104 wks	104	Yes
RTX + CYC followed by belimumab (CALIBRATE)^24^	2021	43	RTX + CYC with or without belimumab	Safety at 48 wks	96	Yes
VOC (AURORA-1)^21^^a^	2021	357	Standard therapy (MMF) + voclosporin 23.7 mg or placebo	CRR at 52 wks	52	Yes
ANF (TULIP-LN)^15^	2022	147	Standard therapy (MMF) + anifrolumab 300 mg, anifrolumab 900 mg first 3 doses followed by 300 mg, or placebo	Change in geometric mean for 24 h UPCR at 52 wks	52	No
OBI (NOBILITY)^23^^a^	2022	125	Standard therapy (MMF) + obinutuzumab 1000 mg or placebo	CRR at 52 wks	104	Yes
TAC^20^	2022	314	Standard therapy (CYC) vs. tacrolimus for initial therapy /Noninferiority trial	CRR, PRR at 24 wks	24	Yes
BI655064^22^	2023	121	Standard therapy (MMF) + BI655064 120 mg, 180 mg, 240 mg, or placebo	CRR at 52 wks	52	No
OBI (REGENCY)^17^^a^	2025	271	Standard therapy (MMF) + obinutuzumab 1000 mg or placebo	CRR at 76 wks	Ongoing	Yes

ABA, abatacept; ANF, anifrolumab; AZA, azathioprine; BEL, belimumab; CRR, complete renal response; CYC, cyclophosphamide; GC, glucocorticoid; LN, lupus nephritis; MMF, mycophenolate mofetil; NRR, nonresponder rate; OBI, obinutuzumab; OCR, ocrelizumab; PERR, primary efficacy renal response; PRR, partial renal response; RTX, rituximab; SLEDAI-2K, Systemic Lupus Erythematosus Disease Activity Index 2000; TAC, tacrolimus; TTT, Thai Tacrolimus Trial; UPCR, urine protein-to-creatinine ratio; VOC, voclosporin.

a These trials demonstrated superiority of the experimental arm over the control arm.

Thirteen trials evaluated the efficacy of an agent in addition to ST; 2 studied the effect of tacrolimus in place of ST as an initial therapy: 1 was a noninferiority trial of tacrolimus versus i.v. cyclophosphamide,^20^ and the other, compared the efficacy of tacrolimus with oral mycophenolate mofetil.^14^ Both of these trials were open label, as was the CALIBRATE trial that investigated the use of cyclophosphamide and rituximab with or without belimumab in refractory LN. The remaining were blinded, placebo-controlled trials (12/15, 80.0%).

### Characteristics of Trial Participants

#### Key Eligibility Criteria

Eligible participants had active LN, defined in part by a proteinuria threshold (1–1.5 g/g for all trials, except for 1 abatacept RCT,^19^ which used a lower threshold of > 0.44 g/g). All trials required biopsy-confirmed LN, with a wide range provided for biopsy timing before screening or enrollment or randomization. Most trials (10/15; 66.7%) required biopsies within 3 to 6 months of screening, whereas 5 studies (33.3%) allowed ≥ 12 months, with additional evidence of worsening urinary or serological parameters, suggesting active disease within the 3 to 6 months before screening. Five RCTs used histological activity as a criterion for inclusion, and 2 trials excluded patients with biopsies demonstrating > 50% glomeruli with sclerosis or fibrosis.

Minimum kidney function thresholds were established as inclusion criteria for almost all trials with eGFR ranging from 20 (CALIBRATE) to 45 ml/min per 1.73 m^2^ (voclosporin trials). Most studies used a <30 ml/min per 1.73 m^2^ threshold for exclusion.

#### Key Participant Characteristics

The median average baseline eGFR across 12 trials was 95 ml/min per 1.73 m^2^. Only the ACCESS trial reported an average eGFR < 90 ml/min per 1.73 m^2^. Four trials included participants with baseline eGFR > 100 ml/min per 1.73 m^2^. Average baseline urine protein-to-creatinine ratio (UPCR) was 3 to 4 g/g. LN duration before trial entry was variably reported; in some studies, such as abatacept,^19^ belimumab,^18^ and obinutuzumab^17^ trials, participants with a recent LN diagnosis undergoing their first full course of initial therapy comprised a majority of the study population. Eleven trials reported the inclusion of participants of > 1 race; non-White participants comprised 48% to 67% (median 57%) of these trial populations. Details are provided in Table 2.

**Table 2:** Study results: characteristics of trial participants and report of efficacy

Investigational treatment (study)	Kidney function	UPCR (g/g)	Duration of LN (mos)	Non-White participants (%)	Efficacy result (%CRR)
OCR (BELONG)^13^	Mean Cr: 0.94 mg/dl	Median: 2.8	Median: 8.4	53	Control: 34.7% OCR 400 mg: 42.7% OCR 1000 mg: 31.5%
ABA^19^	Median Cr: 0.8−0.9 mg/dl	Mean: 3.93 Median: 2.5−3.0	Median duration of current flare: 1	63	Control: 8% ABA 30 mg/kg: 9.1% ABA 10 mg/kg: 11.1%
ABA (ACCESS)^16^	Mean eGFR: 61 ml/min per 1.73 m^2^ Mean Cr: 1.25 mg/dl	Mean: 3.9	Proportion > 12 mos: 29%	50	Control: 31% ABA: 33%
BIIB023 (ATLAS)^25^	Not reported	Not reported	Not reported	Not reported	%CRR+PRR Control: 25% 3 mg/kg: 16% 20 mg/kg: 31%
TAC (TTT)^14^	Mean eGFR: 97 ml/min per 1.73 m^2^ Mean Cr: 0.85 mg/dl	Median: Control: 2.9 TAC: 3.4	Median: 12	N/A^a^	Control: 57.1% TAC: 46.3%
ABA (ALLURE)^26^	Mean eGFR: 95 ml/min per 1.73 m^2^ Mean Cr: 0.93 mg/dl	Mean: 3.78	Median: 9 Mean: 38	Not reported	Control: 33.5% ABA: 35.1%
VOC (AURA-LV)^12^	Mean eGFR: 99.8 ml/min per 1.73 m^2^	Mean: 4.69	Mean: 44.4	59	Control: 19.3% VOC 23.7 mg: 32.6% VOC 39.5 mg: 27.3%
BEL (BLISS-LN)^18^^b^	Mean eGFR: 100.5 ml/min per 1.73 m^2^	Mean: 3.4	Median: 2.4	67	Control: 32% BEL: 43%
RTX + CYC followed by BEL (CALIBRATE)^24^	Mean eGFR: 90.9 ml/min per 1.73 m^2^ Mean Cr: 1.03 mg/dl	Mean: 3.4 Median: 3.1	Proportion >12 months: 84%	63	Control: 32% BEL: 38%
VOC (AURORA-1)^21^	Mean eGFR: 91.3 ml/min per 1.73 m^2^	Mean: 4	Mean: 56	64	Control: 23% VOC: 41%
ANF (TULIP-LN)^15^	Mean eGFR: 93.9 ml/minper 1.73 m^2^	Mean: 3.3	Median: ANF: 6.8 Control: 15.7	54	Control: 31.1% ANF (combined doses): 31%
OBI (NOBILITY)^23^	Mean eGFR: 102 ml/min per 1.73 m^2^ Mean Cr: 0.84 mg/dl	Mean: 3.1	Prior history of LN: 51.5%	57	Control: 23% OBI: 35%
TAC^20^	Mean eGFR: 101.3 ml/min per 1.73 m^2^ Mean Cr: 0.83 mg/dl	Mean: 5.59 g (not normalized to urine Cr)	Mean: 16.8	N/A^a^	Control: 36.3% TAC: 49.6%
BI655064^22^	Mean eGFR: 90.9 ml/min per 1.73 m^2^	Mean: 3.3	Proportion ≥ 6 mos: 60%	48	Control: 48.3% BI655064 120 mg: 38.3% BI655064 180 mg: 45.0% BI655064 240 mg: 44.6%
OBI (REGENCY)^17^	Mean eGFR: 102 ml/min per 1.73 m^2^ Mean Cr: 0.86 mg/dl	Mean: 3.3	Prior history of LN: 57.9%	52	Control: 33.1% OBI: 46.4%

ABA, abatacept; ANF, anifrolumab; BEL, belimumab; Cr, creatinine; CRR, complete renal response; CYC, cyclophosphamide; eGFR, estimated glomerular filtration rate; LN, lupus nephritis; N/A, not applicable; OBI, obinutuzumab; OCR, ocrelizumab; PRR, partial renal response; RTX, rituximab; TAC, tacrolimus; TTT, Thai Tacrolimus Trial; UPCR, urine protein-to-creatinine ratio; VOC, voclosporin.

a Not applicable as national, mono-ethnic trial.

b Primary efficacy renal response (PERR) reported.

### End Points

All studies defined complete renal response (CRR) as an end point: the definitions used are shown in Table 3. Twelve studies defined CRR as the primary end point. Following the 2006 American College of Rheumatology SLE response criteria, all efficacy end points included components of kidney function (defined by eGFR in 11 trials, creatinine in 4) and proteinuria.^27^ Three trials (20.0%) incorporated urinary sediment data.^28^ Five trials (33.3%) explicitly required confirmation of CRR by repeat measurement.

**Table 3:** Definitions of CRR by trial

Investigational treatment (study)	Time to efficacy end point (wks)	Proteinuria threshold (UPCR, g/g)	Change in kidney function	Other	GC limits
OCR (BELONG)^13^	48	0.5	Cr normal AND stable (≤ 125% of baseline^a^)		Not exceeding protocol for treatment of flare
ABA^19^	52	0.26	eGFR normal (≥ 90 ml/min per 1.73 m^2^) if normal at screening visit OR ≥ 90% of *preflare* baseline if screening eGFR < 90	CRR confirmation, Inactive UA sediment	No
ABA (ACCESS)^16^	24	0.5	Cr normal (≤ 1.2 mg/dl) OR stable (≤ 125% of baseline)		Prednisone to 10 mg/d by week 12
BIIB023 (ATLAS)^25^	52	0.5	eGFR normal (unspecified)		No / not available
TAC (TTT)^14^^b^	52	0.5	Cr stable (“return to previous baseline”)		No
ABA (ALLURE)2^5^	52	0.5	eGFR normal (unspecified) OR stable (≥ 85% of baseline)	CRR confirmation, Inactive UA sediment	GC dose not > 10 mg prednisone/d ≥ 28 d before assessment unless GC increase unrelated to kidney disease
VOC (AURA-LV)^12^	24	0.5	eGFR > 60 ml/min per 1.73 m^2^ OR stable (> 80% of baseline)	CRR confirmation	GC dose not > 10 mg prednisone for > 3 consecutive d or 7 total d between weeks 16 and 26^c^
BEL (BLISS-LN)^18^^d^	104	0.7	eGFR ≥ 60 ml/min per 1.73 m^2^ OR stable (≥ 80% of *preflare* baseline)	CRR confirmation	≤ 10 mg/d by week 24^e^
RTX + CYC followed by BEL (CALIBRATE)^24^^b^	48	0.5	eGFR ≥ 120 ml/min per 1.73 m^2^ OR stable (> 80% of baseline) if baseline eGFR < 120		Must adhere to GC protocol
VOC (AURORA-1)^21^	52	0.5	eGFR ≥ 60 ml/min per 1.73 m^2^ OR stable (≥ 80% of *preflare* baseline)	CRR confirmation	GC dose not > 10 mg prednisone for > 3 consecutive d or 7 total d between weeks 44 and 52^c^
ANF (TULIP-LN)^15^^b^	52	0.7	eGFR ≥ 60 ml/min per 1.73 m^2^ OR stable (≥ 0% of baseline)		Must taper to ≤ 15 mg/d by week 12 or < 15 mg/d by week 24. No more than 1 additional steroid pulse; no pulse therapy beyond week 8. GC burst cannot extend beyond week 40
OBI (NOBILITY)^23^	52	0.5	Cr ≤ ULN AND stable (≥ 115% of baseline)	Inactive UA sediment	Cannot receive rescue pulse-dose steroids equivalent to ≥ 500 mg methylprednisolone
TAC^20^	24	0.5	Cr ≤ ULN OR stable (≤ 115% of baseline)	Serum albumin ≥ 3.5 g/dl	Cannot receive ≥1 course of rescue pulse-dose steroids
BI655064^22^	52	0.5	eGFR ≥ 90 ml/min per 1.73 m^2^ OR stable (> 80% of baseline) if week 52 eGFR < 90 ml/min per 1.73 m^2^		Taper to ≤ 10 mg by week 13; however, may increase steroids with flare
OBI (REGENCY)^17^	76	0.5	eGFR ≥ 85% of baseline value (2009 CKD-EPI)		Administration of any of the following from week 64 onward was considered rescue therapy: Methylprednisolone ≥100 mg i.v. or equivalent and/or prednisone ≥ 20 mg/d or equivalent (mean dose) over any 2-wk period

ABA, abatacept; ANF, anifrolumab; BEL, belimumab; CKD-EPI, Chronic Kidney Disease-Epidemiology Collaboration equation; Cr, creatinine; CRR, complete renal response; CYC, cyclophosphamide; eGFR, estimated glomerular filtration rate; GC, glucocorticoid; LN, lupus nephritis; OBI, obinutuzumab; OCR, ocrelizumab; RTX, rituximab; TAC, tacrolimus; TTT, Thai Tacrolimus Trial; UA, urinalysis; ULN, upper limit of normal; UPCR, urine protein-to-creatinine ratio; VOC, voclosporin.

a Baseline refers to screening baseline unless specified as preflare.

b CRR not primary end point.

c AURA LV and AURORA-1: participants did not receive >10 mg prednisone/d for ≥3 consecutive d or for ≥7 d in total during the weeks before the renal response assessment.

d Primary efficacy renal response definition.

e BLISS-LN: did not allow for GC pulse as a result of renal flare.

### Kidney Function

Although the kidney function criteria for clinical remission universally adhered to a maximum of 25% worsening in eGFR or serum creatinine compared with baseline—consistent with the 2006 American College of Rheumatology definition of “stable” kidney function and slightly more conservative than the 2012 National Kidney Foundation/US Food and Drug Administration surrogate marker of persistent 30% to 40% decline in eGFR—exact definitions were heterogenous within these confines.^27^^,^^29^ The strictest definitions required stable and normalized eGFR or creatinine; some studies required eGFR or creatinine to be within the normal range (although not necessarily stable) or to either be above a certain threshold *or* stable; the less stringent definitions called for stability only. eGFR threshold values, when used, ranged from 60 to 120 ml/min per 1.73 m^2^. Two trials (13.3%) compared eGFR or creatinine values at the primary efficacy time point with the more conservative preflare values, whereas the rest did not specify baseline definitions, or used the last eGFR or creatinine measurements before the first day of trial treatment, which could encompass kidney function values during LN flare.^18^^,^^19^^,^^21^

#### Proteinuria

Proteinuria thresholds for CRR were consistent across trials. Thirteen trials used UPCR < 0.5 g/g as the standard for CRR, aligning with the Kidney Disease: Improving Global Outcomes and the European League Against Rheumatism / European Renal Association–European Dialysis and Transplant Association guidelines.^30^^,^^31^ The abatacept trial^19^ used the most conservative UPCR threshold of < 0.26 g/g, which may have been adapted from the 2006 American College of Rheumatology SLE response criteria of < 0.2 g/g.^27^ TULIP-LN (anifrolumab) applied a < 0.7 g/g threshold, which was used as the primary efficacy renal response primary end point in BLISS-LN (belimumab). Notably, the BLISS-LN trial was the only trial that specified the use of spot urine samples for proteinuria measurement. Although timed 24-hour urine collections are the gold standard for quantification of proteinuria, there is strong evidence to suggest that the accuracy of spot measurements is comparable in subnephrotic-range (< 3.5 g/g) proteinuria.^32^ In the Kidney Disease: Improving Global Outcomes 2024 guidelines, spot measurements have eclipsed timed collections as the preferred UPCR method for initial proteinuria evaluation, because they are less prone to user-collection error.

#### Time to Primary End Point

Eight trials (53.0%) assessed the primary end point after 52 weeks (range: 24–104 weeks; Table 1). Eight trials assessed the primary end point at the end of the trial duration, whereas others chose a midpoint for the primary analysis and reported end-of-trial efficacy outcomes as secondary end points. The most common metric reported for efficacy was the absolute difference in the proportion of patients achieving renal response between the trial and control arms, with odds ratios occasionally reported. CRR in the control arms, all of which consisted of ST, with or without placebo, varied from 8% in the 2014 abatacept trial^19^ to 57.1% in the Thai Tacrolimus Trial. Control arm response rates at the primary end point had a mean of 31.2% (SD: 11.5%) across all studies (Table 2). Trials with positive outcomes reported absolute differences in CRR between experimental and control arms ranging from 11% to 18%.

#### Glucocorticoid Use

Although some trials were explicit about glucocorticoid dose and time constraints—e.g., maximum number of consecutive days or use up to a certain time point within the trial—for steroids treating renal or extrarenal flare, restrictions employed across trials were disparate (Supplementary Table S4). Nine trials (60.0%) had rules surrounding rescue steroid use and factored adherence to these rules into the determination of response. When present, steroid rescue limitations spanned from a maximum of 60 mg/d for 2 weeks if nephritis flare (NOBILITY and REGENCY) to immediate classification as nonresponse with experience of renal flare (CALIBRATE) or even discontinuation from the trial if any deviations from steroid protocols or 30% worsening of kidney function (TULIP-LN). Nine trials reported glucocorticoid-related outcomes, including mean daily oral prednisone dose, proportion of patients achieving sustained taper to a prespecified final prednisone dose, and mean cumulative corticosteroid exposure. Nearly all the trials initiated therapy with 1 to 3 days of pulse methylprednisolone, followed by weight-based oral steroid dosing, with maximum doses of 25 to 60 mg of prednisone daily. Trends show declining maximum prednisone dose over time, with earlier trials using 60 mg/d and more recent trials initiating ≤ 45 mg/d. The voclosporin trials used a low-starting prednisone dose (25 mg/d) with taper periods of 10 to 24 weeks, maintaining a daily dose of 2.5 to 10 mg.

#### Secondary End Points

Common secondary end points included rates of partial response and changes in clinical indices such as the British Isles Lupus Assessment Group index or SLE Disease Activity Index. Partial renal response generally required improvement in UPCR to ≤ 50% of baseline. Other frequently reported outcomes were time to renal response, rates of sustained response, rates of combined complete and partial response, and efficacy at other time points. Two studies evaluated rates of LN flare and 5 compared patient-reported outcomes between treatment arms.

### Characteristics of Positive Trials

Five of the 15 trials demonstrated superiority of the experimental arm over the control arm. These trials investigated the efficacy of voclosporin, belimumab, and obinutuzumab compared with ST. The investigational treatments had modest effect compared with the control arm, with numbers needed to treat ranging from 5.5 to 9 (experimental therapy must be added to ST regimen for 5.5–9 patients to achieve 1 additional CRR). Commonalities shared among these positive trials include later publication date, baseline eGFR ≥ 90 ml/min per 1.73 m^2^, and industry sponsorship. Four of the 5 positive studies (REGENCY, NOBILITY, BLISS-LN, and AURA-LV) had average baseline eGFR ≥ 99 ml/min per 1.73 m^2^, the highest averages among the included studies. Apart from REGENCY and NOBILITY, all the trials employed renal response criteria of eGFR ≥ 80% of baseline or > 60 ml/min per 1.73 m^2^.

### ROB

Four trials (26.7%) were graded as having high ROB with respect to assessment of efficacy-related outcomes (Supplementary Table S3). Because some trials did not specify such outcomes as their primary objectives, the bias judgement does not necessarily represent the overall quality of the trial. The most common reason for high bias was handling of missing data, including strict criteria for participant discontinuation and premature trial termination. A detailed discussion of the ROB is presented in the Supplementary Methods.

## Discussion

### Evolution of End Points in Clinical Trials of Chronic Kidney Disease as a Guide for End Points in LN Trials

**Figure 1:**
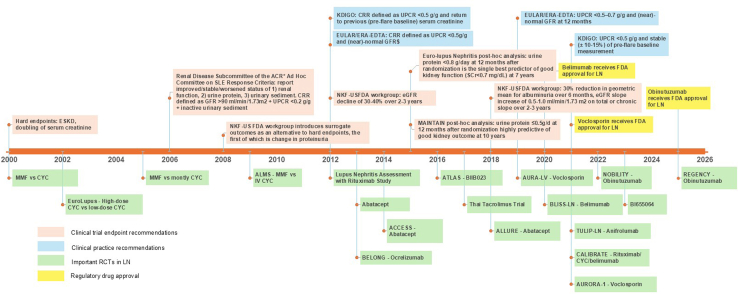
Recommendations for end points used in clinical trials of LN through the years. ACR, American College of Rheumatology; CRR, complete renal response; CYC, cyclophosphamide; EDTA, European Dialysis and Transplant Association; eGFR, estimated glomerular filtration rate; ERA, European Renal Association; ESKD, end-stage kidney disease; EULAR, European League Against Rheumatism; FDA, US Food and Drug Administration; GFR, glomerular filtration rate; KDIGO, Kidney Disease: Improving Global Outcomes; LN, lupus nephritis; MMF, mycophenolate mofetil; NKF, National Kidney Foundation; RCT, randomized control trial; SLE, systemic lupus erythematosus; UPCR, urine protein-to-creatinine ratio.

The increase in pharmaceutical trials dedicated to LN is partly driven by the evolution of clinical end points meeting requirements for regulatory approval (Figure 1). Previously, clinical trials in chronic kidney disease (CKD) employed end points capturing only late-stage features (“hard end points”).^33^, ^34^, ^35^ However, use of such long-term outcomes to gauge treatment efficacy substantially lowers the sensitivity in identifying treatments that make a difference in clinical outcomes and increases trial cost. Regulatory acceptance of surrogate outcomes has been critical in increasing the feasibility of trials, enabling reductions in follow-up time and effect size. Recommendations from scientific workshops cosponsored by the National Kidney Foundation; the US Food and Drug Administration^36^; and organizations with clinical authority such as Kidney Disease: Improving Global Outcomes, American College of Rheumatology, European League Against Rheumatism, and the European Renal Association, have the most strongly driven end point choice.

Although surrogate outcomes have improved access to potentially beneficial treatments, they cannot replace the ultimate goal of long-term follow-up of trial participants to evaluate treatment efficacy and its correlation with long-term kidney outcomes. Longitudinal data from the Euro-LN and MAINTAIN cohorts have critically influenced end point guidelines, underscoring the importance of proteinuria at the 12-month mark and reducing emphasis on the role of urinary red blood cells in long-term prognostication; although this simply may be because of operational issues such as collection and visualization of fresh urine specimens.^28^^,^^37^

### Commentary on Clinical Trials of LN

#### End Points

Recent trials have adhered to guidelines in defining primary outcomes for target proteinuria. However, kidney function end points remain heterogeneous because of vague clinical recommendations for defining CRR such as return to preflare baseline (Kidney Disease: Improving Global Outcomes) or achievement of “near-normal” eGFR (European League Against Rheumatism / European Renal Association). Because preflare baseline eGFR is difficult to establish in the context of a clinical trial, this lofty goal often transforms into a standard for stable kidney function compared with values obtained at randomization or screening, which are usually at the time of a flare. The BLISS-LN and Abatacept^19^ trials aimed to use a CRR definition based on preflare values, assigned using a single serum creatinine measurement taken before the participants’ current flare; however, it is unlikely that a single measurement is reflective of participants’ true kidney function and all participants have sufficient baseline laboratory information for a preflare determination. Thus, this standard is untenable for most trials.

Defining “near-normal” eGFR is problematic. Many trials use > 60 ml/min per 1.73 m^2^ to define CRR, which is exceedingly low considering that the median baseline eGFR for nearly all recent trials was > 90 ml/min per 1.73 m^2^. The ability to determine kidney function response to treatment is nearly eliminated when studies use a composite definition incorporating a low absolute eGFR threshold (e.g., eGFR stable odds ratio > 60 ml/min per 1.73 m^2^). Stability or improvement as measured by eGFR slope (stratified by baseline CKD stage) may better capture therapeutic differences while controlling for variations in baseline kidney function. Until published CRR guidelines improve, studies using composite end points can better reflect therapeutic benefit by reporting the percentage of CRR cases driven by each end point component. Because CRR rates are often driven by UPCR, and 12-month proteinuria remains a poor predictor of long-term kidney outcomes (negative predictive value: 0.67, 95% confidence interval: 0.46–0.83), better markers are needed to differentiate patients who will develop advanced CKD. Evidence suggests that one-third of patients experience progressive eGFR decline (> 5 ml/min per 1.73 m^2^/yr) despite achieving CRR at 1 year. This does not bode well for long-term avoidance of ESKD because many patients with LN are diagnosed in their third decade of life.^38^

Recent end point definitions integrate glucocorticoid limits in CRR, which should become standard practice in clinical trials. Although glucocorticoids play an important role in managing LN, their adverse effects are associated with exposure at peak and cumulative doses.^39^ Future trial guidelines should define glucocorticoid thresholds for treatment failure because liberal use increases the risk of adverse effects and masks therapeutic differences between treatment arms. In addition, clinical trials often use higher glucocorticoid doses than those in real-world clinical settings of LN, which may inflate response rates in the trial setting.^40^ Although most recent trials regulate glucocorticoid use, criteria vary; some trials classify patients as complete or partial responders, despite receiving high steroid doses. Universal reporting of weight-adjusted total cumulative glucocorticoid dose and total duration on high-dose steroids should be implemented as a minimum for assessing treatment efficacy and risk of adverse effects.^39^ Measurement tools such as the Glucocorticoid Toxicity Index can invaluably contribute to shaping guidelines for glucocorticoid use.^41^

Another focal point in the clinical trial landscape is the use of patient-reported measures of efficacy; these patient-reported outcomes remain underused, despite the overall goal of improving health-related quality of life for people with LN. Trials should be required to report patient-reported outcomes as secondary or exploratory outcomes. In addition, there is a need for a standardized, validated, lupus-specific, health-related quality of life index to enable comparison across trials. Further research in collaboration with patients is required for the development of LN-specific instruments.

#### Eligibility Criteria

All trials included in our SLR aimed to enroll participants with active LN. *Post hoc* analysis of the BELONG trial, which defined activity using UPCR ≥ 1 g/g and required biopsy-proven class III or IV nephritis within 6 months of randomization (per local pathology read), showed that 11 of the 70 biopsies reviewed would not have met the inclusion criteria for the trial on the basis of LN class and/or activity. Despite various approaches to capturing active disease, the reality is that conventional markers, such as anti–double-stranded DNA antibodies, complement levels, and proteinuria lack sensitivity and specificity in detecting LN flare.^42^ In particular, proteinuria is a marker of damage and activity. Including participants without class III/IV LN or with only chronic biopsy changes introduces nondifferential misclassification, which biases toward a null effect of investigational treatment over placebo. Investigators can mitigate this by employing a central reading agency to determine histological eligibility. Routine reporting of the quality of biopsies (e.g., % of screening biopsies with > 10 glomeruli) should be required to convey the validity of eligibility assessments.

Nearly every trial reviewed excluded patients with severely impaired kidney function. The reasons may be related to safety (if the treatment is renally excreted) or practical (such as heightened likelihood of ESKD or death), which could negatively affect trial outcomes because of decreased follow-up time, thus impairing the ability to assess treatment efficacy and safety. However, patients with severe disease may benefit most from new therapies and the results may provide valuable insights into LN pathophysiology. Importantly, though it may be reasonable to exclude a patient with > 50% glomerular or interstitial fibrosis and atrophy on their biopsy, it is not reasonable to exclude a patient with a reduced eGFR secondary only to severe acute LN or nephrotic state with no chronicity. Almost all trials included in this review would have excluded both sets of patients. An SLR assessing theoretical eligibility of patients with LN in a tertiary hospital cohort for 33 recent RCTs concluded that up to one-third of the cohort would have fulfilled the exclusion criteria because of disease severity or use of prohibited baseline immunosuppressive treatments.^43^ This suggests poor external validity of RCTs in LN, bringing into question efficacy rates of therapies when applied to real-world populations. Furthermore, only a minority of trials report screen failure rates, and publication of reasons for ineligibility is even more rare.^43^ Such information is crucial to understanding to whom the trial results apply and for targeting ways to increase the enrollment of underrepresented subgroups. Protocols addressing exclusions based on previous therapy and control arm treatments will need major restructuring in the next few years because changes to ST regimens are applied to clinical practice. In addition, as more treatments are approved for LN, it will become increasingly important to ensure washout periods for each agent are standardized across trials.

#### Future Directions

LN research and therapy are at a pivotal stage, with breakthroughs in pathophysiology and momentum from new treatment approvals driving interest in new therapies. Currently, 12 registered phase 2/3 RCTs are underway (Table 4). However, our understanding of disease processes, particularly at an individual level, and our ability to predict ESKD progression, remain limited. Well-controlled, comprehensively reported exploratory trials, with reliable inclusion and exclusion criteria are needed to recruit populations representative of the real-world experience. Until reliable prognostic biomarkers emerge, standardizing trial design and reporting will improve assessment of therapies.

**Table 4:** Registered RCTs assessing initial therapy for LN and their proposed CRR definitions

Investigational treatment/sponsor	Study identifier	Time to efficacy end point (wks)	Proteinuria threshold (UPCR, g/g)	Change in kidney function	GC limits	Status/ estimated primary study completion
Zanubrutinib/ BeiGene	NCT04643470	49	-	-	-	Completed/ 2024
Iptacopan/Novartis Pharmaceuticals	NCT05268289	24	0.5	eGFR ≥ 90 ml/min per 1.73 m^2^ or ≥ 85% of baseline	-	Recruiting/ 2024
Efgartigimod i.v. / Argenx	NCT05810948	24	Change in UPCR from baseline	-	-	Completed/ 2025
Telitacicept/ RemeGen Co, Ltd	NCT05680480	48	0.5	eGFR ≥ 60 ml/min per 1.73 m^2^ or ≥ 80% of baseline	-	Estimated completion/ 2026
Mesenchymal stromal cells/ Universidad de los Andes, Chile	NCT03917797	-	0.5	eGFR ≥ 120 ml/min per 1.73 m^2^ or ≥ 80% of baseline	Prednisone dose ≤ 10 mg/d	Unknown status, /Estimated completion 2025
ANF (IRIS)/ AstraZeneca	NCT05138133	52	0.5	eGFR ≥ 60 ml/min per 1.73 m^2^ or > 80% of baseline	-	Recruiting / 2026
Atacicept (COMPASS)/ Vera Therapeutics, Inc.	NCT05609812	52	0.5	eGFR ≥ 60 ml/min per 1.73 m^2^ or, if baseline < 60 ml/min per 1.73 m^2^, then ≥ 80% of baseline	-	Suspended / 2026
VIB4920 (VIBRANT)/ National Institute of Allergy and Infectious Diseases	NCT05201469	36	0.5	eGFR ≥ 120 ml/min per 1.73 m^2^ or, if < 120 ml/min per 1.73 m^2^, then ≥ 80% of baseline eGFR	Prednisone ≤ 5 mg by week 8	Recruiting / 2026
OBI in adolescents (POSTERITY)/ F. Hoffmann-La Roche	NCT05039619	76	0.5	eGFR ≥ 85% of baseline	-	Recruiting/ 2027
Ianalumab (SIRIUS-LN)/ Novartis Pharmaceuticals	NCT05126277	72	0.5	eGFR ≥ 90 ml/min per 1.73 m^2^ or ≥ 85% of baseline	-	Recruiting / 2027
VOC/ Qilu Pharmaceutical Co., Ltd.	NCT06406205	52	0.5	eGFR ≥ 60 ml/min per 1.73 m^2^ or confirmed ≥ 80% of baseline	-	Recruiting / 2027
Nipocalimab/Janssen Research and Development, LLC	NCT04883619	52	-	eGFR ≥ 120 ml/min per 1.73 m^2^ or ≥ 80% of baseline	-	Active, not yet recruiting / 2028
Anti-CD19 CAR-T cells/ Kyverna Therapeutics	NCT06342960	12, 24, 52	-	-	-	Active, not recruiting/2028
Rapcabtagene autoleucel/ Novartis Pharmaceuticals	NCT06581198	52	0.5	eGFR ≥ 90 ml/min per 1.73 m^2^ or ≥ 85% of baseline	Low-dose GCs week 24 and beyond	Active, recruiting/2032

ANF, anifrolumab; CAR, chimeric antigen receptor; CRR, complete renal response; eGFR, estimated glomerular filtration rate; GC, glucocorticoid; LN, lupus nephritis; OBI, obinutuzumab; RCT, randomized controlled trial; UPCR, urine protein-to-creatinine ratio; VOC, voclosporin.

By capturing larger, more diverse cohorts with standardized follow-up, a well-designed RCT can provide valuable insights into prognostic factors and barriers to care. Data collection should include novel biomarkers linked to disease activity, CKD progression, and response to therapy, using quality of life instruments adapted for kidney diseases. In addition, posttreatment biopsy protocols must be refined, because current methods for evaluating treatment efficacy and predicting prognosis are suboptimal. Furthermore, numerous methods, such as logistical support and detailed explanation of the benefits, should be employed to encourage study participants undergo repeat kidney biopsies given the profound scientific benefit that could lead to improvements in therapeutic advances. In addition, central biopsy reads would be important because of the discordance between local and central reads that have been observed.^44^ Simultaneously, we must expand participation in trials to improve the generalizability of results and generate data that can eventually guide tailored treatment algorithms. This can be achieved by addressing social determinants of health and socioeconomic status, interviewing potential participants, and publishing data on screen failures to elucidate barriers to trial participation.

#### Recommendations to Facilitate Cross-Trial Comparisons

##### Patient Involvement

Involve patients early in trial codesign from inception to identify and address barriers to trial participation. All trials should report the nature and extent of patient participation and should include patient-reported outcomes.

##### Eligibility

Use central reading of kidney biopsies to determine eligibility. Biopsies should undergo full central evaluation of lesions using the 2018 International Society of Nephrology/Renal Pathology Society classification and activity/chronicity indices, and pictures of immunofluorescence microscopy should be reviewed by central readers for grading of immune complex deposits. Exclude patients based on percentage of glomerular and interstitial scarring and not on eGFR. Report screen failure rates and reasons for failure.

##### End Points

When using a composite primary end point for renal response, report the proportion of participants who met the individual components of the composite outcome to clarify efficacy drivers. Report CKD stages of study participants and proportions of patients with 30% and 40% decrease in eGFR, eGFR slope, and ESKD events. All patients should be offered an optional repeat kidney biopsy coinciding with the time of evaluation of the primary end point in all studies to confirm CRR.

##### Steroid Use

Report median and interquartile range for weight-adjusted cumulative doses of glucocorticoids to which participants were exposed during the trial. Incorporate glucocorticoid limits into the primary end point (clearly define thresholds considered treatment failure). Limits should include definitions for failure of steroid taper, permissible dosing and duration for flare, and time restraints on when participants can receive higher doses of steroids in relation to the primary end point.

##### Data Sharing

The rarity of LN limits individual trial sample sizes for robust subgroup analyses. Although there are regional registries for patients with SLE, having a unified national registry for LN would enhance research on long-term outcomes. An accessible database of clinical trial data would enable meta-analyses and inform biomarker selection for future trials.

## Conclusion

Despite trial limitations, recent research has enhanced our ability to care for patients living with LN. The field is experiencing a renaissance, driven by artificial intelligence and techniques in molecular characterization; over the next decade we will see a rapid expansion in LN knowledge. There is an urgent need for the LN research community and authoritative bodies to establish higher standards for RCT design and reporting to enhance the translation of emerging knowledge into improvement of patients’ lives.

The major limitation of this review is the lack of individual-level data from the included trials to make more meaningful, equivalent comparisons between them. In addition, as described in greater detail in the Supplementary Material, more than half of the studies included in our SLR had some or high levels of ROB.

This SLR was not prospectively registered. Data were collected through prespecified methods and are as complete as possible given published protocols, supplementary materials, and abstracts/manuscripts. Internal trial documents were used as data sources for Roche/Genentech-sponsored trials.

## Disclosure

SG was an employee of Genentech, Inc. during preparation. MAD has received consulting fees from AstraZeneca, Aurinia, Biogen, Genentech, Inc, and GlaxoSmithKline; and reports professional services for Cabaletta, Janssen, and Novartis. RBJ has received research support and/or consulting fees from F. Hoffmann-La Roche Ltd and CSL Vifor. LL has received consulting fees and/or reports professional services for Alexion, Argenx, AstraZeneca, Boehringer Ingelheim, Carna Health, F. Hoffmann-La Roche Ltd, GlaxoSmithKline, Kezar, Nkarta, Novartis, Otsuka, and Pfizer. JA is the Chair of LUPUS Europe. EM is an employee and shareholder of F. Hoffmann-La Roche Ltd. JART and WFP are employees of Genentech, Inc. and shareholders of F. Hoffmann-La Roche Ltd. AT was an employee and shareholder of F. Hoffmann-La Roche Ltd at the time of analysis. AM has received consulting fees and/or reports professional services for BMS, F. Hoffmann-La Roche Ltd, GlaxoSmithKline, Kezar, Novartis, and Pfizer.

## Supplementary Material

Supplementary File (PDF) **Supplementary Methods. Figure S1.** PRISMA flow chart for trial selection of the SLR. **Table S1.** Search terminology for online databases/registries used in the SLR. **Table S2.** Details of trial data extraction for SLR. **Table S3.** ROB in included studies. **Table S4.** GC-related trial details. PRISMA checklist.

## Footnotes

Supplementary File (PDF)

**Supplementary Methods.**
**Figure S1.** PRISMA flow chart for trial selection of the SLR. **Table S1.** Search terminology for online databases/registries used in the SLR. **Table S2.** Details of trial data extraction for SLR. **Table S3.** ROB in included studies. **Table S4.** GC-related trial details. PRISMA checklist.
