# Supplementary material for: Systematic Review of Trial Design and End Points in Lupus Nephritis
Source: Kidney Int Rep. 2026 Mar 10;11(5):106479. doi: 10.1016/j.ekir.2026.106479 (PMC13090550; doi:10.1016/j.ekir.2026.106479)
Supplement: Supplementary File (PDF) — Supplementary Methods. Figure S1. PRISMA flow chart for trial selection of the SLR. Table S1. Search terminology for online databases/registries used in the SLR. Table S2. Details of trial data extraction for SLR. Table S3. ROB in included studies. Table S4. GC-related trial details. PRISMA checklist. [file mmc1.pdf]

Systematic review of trial design and endpoints in lupus nephritis

Sophia Giang, MD[12]*, Maria A. Dall'Era, MD[2], Rachel B. Jones, MD [3], Liz Lightstone, MD[4], Jeanette Andersen, BA[5], Elsa Martins, Pham. D, PhD[6], Jorge A. Ross Terres, MD[1], Armando Turchetta, MD[7]*, William F. Pendergraft III, MD, PhD[1], Ana Malvar, MD[8]

*Affiliation at the time of analysis.

[1]Genentech, Inc., South San Francisco, CA, USA; [2]Division of Pediatric Nephrology, Stanford Children's Health, Palo Alto, CA, USA; [3]Cambridge University Hospitals NHS Foundation Trust, Cambridge, UK; [4]Department of Immunology and Inflammation, Imperial College London, London, UK; [5]Lupus Europe, Brussels, Belgium; [6]F. Hoffmann-La Roche Ltd, Basel, Switzerland; [7]Hoffmann-La Roche Ltd, Mississauga, ON, Canada; [8]Nephrology Unit, Hospital Fernández, Buenos Aires, Argentina

Corresponding author

Ana Malvar

Address: 2652 Calle Bulnes, Ciudad Autónoma de Buenos Aires C1425DKV, Argentina

Email: avmperrin@yahoo.com.ar

Phone/Fax number: +54 9 1159819591

Supplementary Methods

Inclusion and exclusion criteria were defined for included studies by SG, EM, WP, JART, and AT. EMBASE, PubMed®, Cochrane Library and ClinicalTrials.gov were searched for studies published between Jan 2013 and Jul 2023 with a later update of the end date to Feb 2025. The major search terminology utilized were ‘lupus nephritis’, ‘randomized controlled trial’, ‘interventional/drug trial’; detailed search methodology is documented in **online Supplemental Table 1**. SG performed the search and screened results for qualifying studies. Five authors developed the methodology and collaboratively reviewed the list of articles. Search results from all databases/registers were imported into a citation manager. Duplicates were initially eliminated using an automated process provided by the citation manager with manual identification of the remaining duplicates. Studies that were questionable in their fulfilment of criteria were jointly reviewed by SG, EM, WP, JART and AT; all authors approved the final list of included studies.

Trial details were extracted by SG, EM and AT using a standardized form. Results were amalgamated by SG, who extracted results and resolved discrepancies by reviewing the primary publication, supplemental documents and protocol.

Supplementary Risk of Bias Discussion

Handling of missing data was a particularly high concern in the event of premature trial termination, which occurred in the BELONG (ocrelizumab) and ATLAS (BIIB023) trials. The overall ROB for ATLAS was graded “high”, mostly due to the fact that the results and methods were only published in the form of an abstract. BELONG defined its modified intention-to-treat population as participants achieving at least 32 weeks of follow-up – the time to receive at least four infusions of the trial treatment. Whilst this may have been logical, it is not clear whether other definitions were considered and whether the choice was dependent upon results. Given the trial was negative, it is unlikely that a posteriori decision significantly affected the efficacy result.

Of note, rates of discontinuation in the TULIP-LN trial were high due to strict criteria for discontinuation, including eGFR decline to 30% of baseline or nephrotic-range proteinuria as early as 12 weeks from the trial start date. As non-responder status is carried forward from trial discontinuation, such strict criteria result in loss of information on longer-term outcomes and may unfairly prevent the use of potentially beneficial therapies. Many of the discontinuations in TULIP-LN were due to use of restricted therapies prior to the trial visit – this applied to 44% of participants in the placebo arm and 25% of participants in the combined anifrolumab arms. These discrepant rates of discontinuation favoring efficacy in the anifrolumab arm are less significant considering the negative findings.

Other marked sources of bias in efficacy results from included studies were the primary report of per protocol efficacy analyses in the Zheng tacrolimus and CALIBRATE studies. Per protocol populations are subject to bias in superiority trials, as they are defined by receipt of treatment/adherence to treatment, which is affected by several factors that favor better clinical outcomes as well as treatment tolerability.

Supplementary Figure S1. PRISMA flow chart for trial selection of the SLR

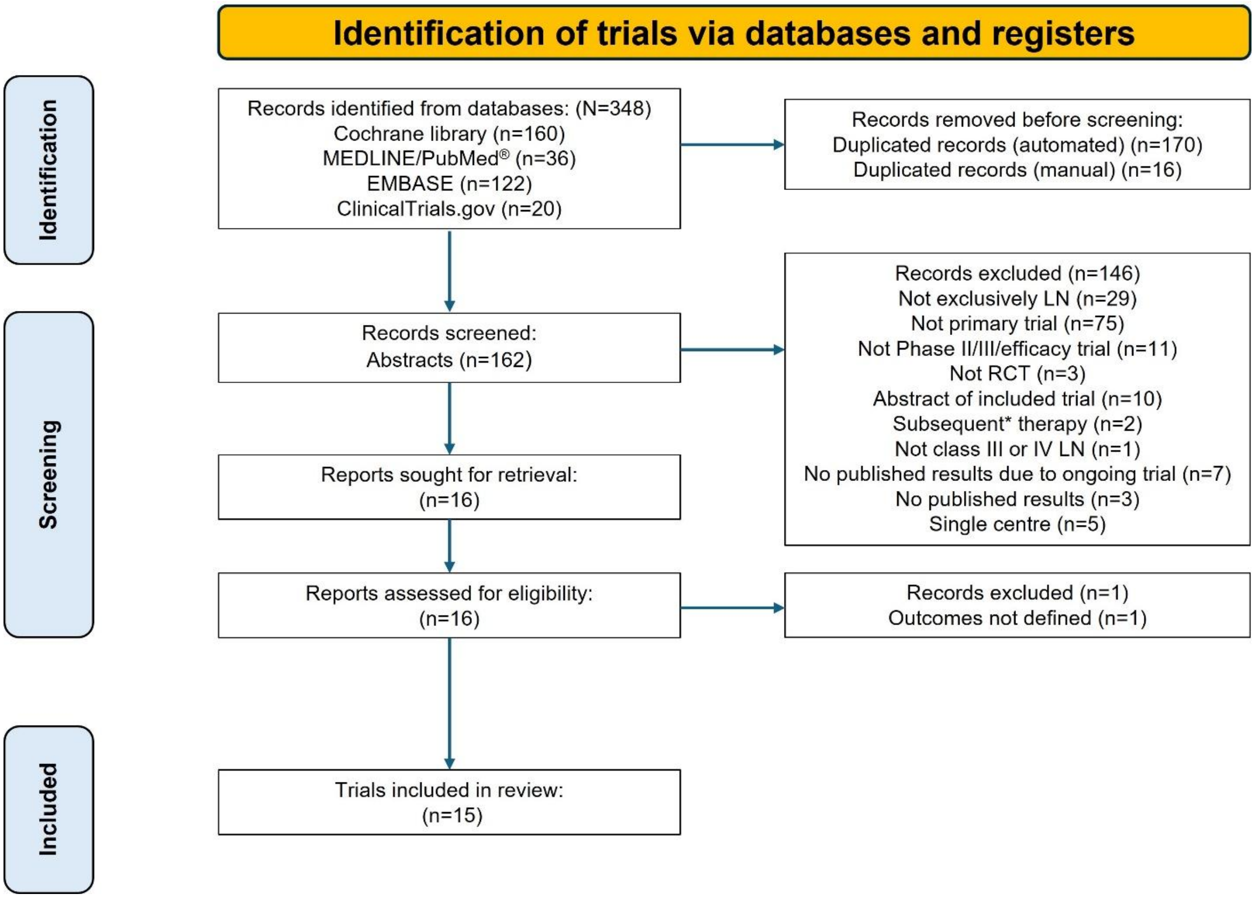

LN, lupus nephritis; PRISMA, Preferred Reporting Items for Systemic reviews and Meta-Analyses; RCT, randomized controlled trial.

Supplementary Table S1. Search terminology for online databases/registries used in the SLR

**Database/register**	
MEDLINE/PubMed®	*Without MeSH terms:* (((lupus nephritis AND (y_10[Filter])) AND ((((study drug) OR (pharmaceutic)) OR (treatment drug)) OR (drug therapy) AND (y_10[Filter]))) AND (((randomized controlled trial) OR (RCT)) OR (randomised controlled study) AND (y_10[Filter]))) AND ((phase 2) OR (phase 3) AND (y_10[Filter]))<br><br>*With MeSH terms:* (("Lupus Nephritis/drug therapy"[Mesh]) AND "Randomized Controlled Trial" [Publication Type]) AND "Clinical Trial, Phase II" [Publication Type]
Cochrane Library	Title Abstract Keyword: lupus nephritis<br>AND All Text: randomized controlled trial OR randomized controlled study<br>AND All Text: drug therapy OR study drug OR pharmaceutical OR drug<br>AND All Text: phase 2 OR phase 3 OR phase II OR phase III
EMBASE	#5 #1 AND #2 AND #3 AND #4<br>#4 (((**phase** AND **ii** OR **phase**) AND **2** OR **phase**) AND **iii** OR **phase**) AND **3**<br>#3 **'article'**/it OR **'conference abstract'**/it<br>#2 **'lupus erythematosus nephritis'**/dm<br>#1 (**'lupus nephritis'**/exp OR **'lupus nephritis'** OR (**lupus** AND (**'nephritis'** OR **'nephritis'**/exp OR **nephritis**))) AND (**pharmaceutical** OR **'drug'** OR **'drug'**/exp OR **drug**) AND (**rct** OR **'randomized controlled trial'**/exp OR **'randomized controlled trial'** OR (**randomized** AND **controlled** AND (**'trial'** OR **'trial'**/exp OR **trial**))) AND ([controlled clinical trial]/lim OR [randomized controlled trial]/lim) AND [2013-2024]/py
ClinicalTrials.gov	Condition/disease: lupus nephritis<br>Study phase: phase 2, phase 3<br>Study Type: Interventional<br>Study Results: With results<br>Date Range: Study completion from 01/01/2013 to 02/07/2025

MeSH, Medical Subject Headings.

Supplementary Table S2. Details of trial data extraction for SLR

TRIAL IDENTIFICATION

- Investigational treatment
- NCT number
- Title of publication
- Authors
- Year of publication
- Trial start date

DESIGN

- Clinical trial phase
- Blinding
- Use of placebo control
- Industry sponsorship
- Duration of follow-up
- Inclusion/exclusion criteria
    - UPCR for inclusion
    - Definition of active LN for inclusion
        - Timing of kidney biopsy
        - Laboratory criteria, if any
- Treatment
    - Investigational treatment regimen
    - Control regimen
    - ST regimen
    - Steroid use
        - Pulse steroid regimen, if any
        - Initial dose of prednisone/equivalent
        - Maximum dose of prednisone/equivalent
        - Timing of taper start
        - Taper duration
        - Final prednisone/equivalent dose
    - Limits on steroid use, if any
- Post-treatment kidney biopsy obtained
- Endpoints
    - Primary outcome definition
        - GFR equation used
        - UPCR method used

▪ Definitions of CRR by renal function change, UPCR threshold, urine sediment, and/or other<br>▪ Use of preflare or screening baseline GFR/creatinine<br>▪ Timing of outcome measurement<br>▪ Incorporation of steroid limits<br>▪ Confirmation of renal response<br>○ Secondary outcomes<br>○ Exploratory outcomes
**OUTCOMES**
● Study population<br>○ Continent(s) included<br>○ Global (2+ continents)<br>○ Number of participants randomized<br>○ Number of participants in modified intention-to-treat population<br>○ Baseline GFR/creatinine<br>○ Baseline UPCR<br>○ Duration of disease<br>● Efficacy results<br>○ Premature termination<br>○ Achieved primary outcome<br>○ Primary efficacy endpoint estimate, with 95% CI<br>○ Steroid-related outcomes

CI, confidence interval; CRR, complete renal response; GFR, glomerular filtration rate; LN, lupus nephritis; ST, standard therapy; UPCR, urine protein-to-creatinine ratio.

Supplementary Table S3. ROB in included studies

Treatment (study)	Randomization	Assigned intervention	Missing outcomes	Measurement of outcome	Reporting	Overall
OCR (BELONG)	Low	Low	Low	Low	Some	Some
ABA	Some	Low	Low	Low	Low	Some
ABA (ACCESS)	Low	Low	Low	Low	Low	Low
BIIB023 (ATLAS)	Some	Low	High	Low	Some	High
TAC (TTT)	Low	Low	Low	Low	High	High
ABA (ALLURE)	Low	Low	Low	Low	Low	Low
VOC (AURA-LV)	Low	Low	Low	Low	Low	Low
BEL (BLISS-LN)	Low	Low	Low	Low	Low	Low
Rituximab + CYC followed by BEL (CALIBRATE)	Low	High	High	Low	High	High
VOC (AURORA-1)	Low	Low	Low	Low	Low	Low
ANF (TULIP-LN)	Low	Low	Some	Low	Low	Some
OBI (NOBILITY)	Low	Low	Low	Low	Low	Low
TAC (Zheng)	Some	High	Low	Low	High	High
BI655064	Low	Low	Some	Low	Low	Some
OBI (REGENCY)	Low	Low	Low	Low	Low	Low

ABA, abatacept; ANF, anifrolumab; AZA, azathioprine; BEL, belimumab; CYC, cyclophosphamide; GC, glucocorticoid; OBI, obinutuzumab; OCR, ocrelizumab; LN, lupus nephritis; ROB, risk of bias; RTX, rituximab; TAC, tacrolimus; TTT, Thai Tacrolimus Trial; VOC, voclosporin.

Supplementary Table S4. GC-related trial details

Investigational treatment (study)	Mandatory initial pulse-dose GC	Initial daily prednisone dose	Week of taper end	Final prednisone dose (mg)	GC-related outcomes reported (other than those included in endpoints)
OCR (BELONG)	No – allowed MP 3 g/day until day 15	0.5–0.75 mg/kg (max 60 mg) x 16 days	10	≤10	Mean daily oral prednisone dose
ABA	Yes – ≤4-week high-dose oral GC prior to randomization or MP 500 mg x 3 doses	0.8 mg/kg (max 60 mg) x 4 weeks	12	≤5	None
ABA (ACCESS)	No – per site PI discretion	60 mg x 2 weeks	12	10	None
BIIB023 (ATLAS)	Yes – MP 500 mg/day x3 days	25–45 mg	12	10	None
Tacrolimus (TTT)	Yes – ≤4-week high-dose oral GC prior to randomization	0.7–1 mg/kg (max 60 mg) x up to 4 weeks prior to randomization	22 (max given range of 5–10 mg taper q2 weeks)	5	None
ABA (ALLURE)	Unclear	0.75 mg/kg (max 60 mg) x 8 weeks	20	10	None
VOC (AURA-LV)	Yes – MP 0.5 g/day x2 doses	0.4 mg/kg (max 25 mg) x 2 weeks	16	2.5	None
BEL (BLISS-LN)	No – per site PI discretion; MP 500 mg–1g x 1–3 doses	0.5–1 mg/kg (max 60 mg) x 2 weeks	24	≤10	Proportion of patients receiving ≤5 mg and ≤7.5 mg/day

RTX + CYC followed by BEL (CALIBRATE)	No	40 mg x unspecified duration	12	10	None
VOC (AURORA-1)	Yes – MP 0.25 or 0.5 g/day x2 doses	20 mg (for persons <45 kg) or 25 mg (≥45 kg) x 2 weeks	16	2.5	None
ANF (TULIP-LN)	Yes – MP 500 mg x 1 dose	0.5 mg/kg (max 40 mg); patients can enter study on this dose	24	≤7.5	Proportion of patients with sustained GC taper, proportion of patients receiving <7.5 mg/day between weeks 24 and 52
OBI (NOBILITY)	Yes – MP 1–3 g total	0.5 mg/kg (max 60 mg) x 15 days	12	7.5	Median and IQR for cumulative GC exposure
TAC (Zheng)	Yes – MP 500 mg x 3 doses	0.8 mg/kg (max 45 mg) x 4 weeks	22	10	None
BI655064	Yes – MP 500 mg x 3 doses (per PI discretion up to 1 g x3 doses)	25–45 mg x 2 weeks	12	10	Mean daily GC dose, reported high GC
OBI (REGENCY)	Yes – MP 1–3 g total	0.5 mg/kg (max 60 mg) x 15 days	12	7.5	Median and IQR for cumulative GC exposure

ABA, abatacept; ANF, anifrolumab; BEL, belimumab; CYC, cyclophosphamide; GC, glucocorticoid; IQR, interquartile range; LN, lupus nephritis; MP, methylprednisolone; OBI, obinutuzumab; OCR, ocrelizumab; PI, Principal Investigator; q2, every 2 weeks; RTX, rituximab; TTT, Thai Tacrolimus Trial; VOC, voclosporin.

PRISMA 2020 Checklist

Section and Topic	Item #	Checklist item	Location where item is reported
**TITLE**			
Title	1	Identify the report as a systematic review.	Page 1
**ABSTRACT**			
Abstract	2	See the PRISMA 2020 for Abstracts checklist.	Page 2
**INTRODUCTION**			
Rationale	3	Describe the rationale for the review in the context of existing knowledge.	Page 3
Objectives	4	Provide an explicit statement of the objective(s) or question(s) the review addresses.	Page 3
**METHODS**			
Eligibility criteria	5	Specify the inclusion and exclusion criteria for the review and how studies were grouped for the syntheses.	Page 3 and Supplementary Fig 1
Information sources	6	Specify all databases, registers, websites, organisations, reference lists and other sources searched or consulted to identify studies. Specify the date when each source was last searched or consulted.	Page 3 and Supplementary Fig 1
Search strategy	7	Present the full search strategies for all databases, registers and websites, including any filters and limits used.	Page 3 and Supplementary Table 1 and 2
Selection process	8	Specify the methods used to decide whether a study met the inclusion criteria of the review, including how many reviewers screened each record and each report retrieved, whether they worked independently, and if applicable, details of automation tools used in the process.	Page 3-4 and Supplementary Table 1 and 2
Data collection process	9	Specify the methods used to collect data from reports, including how many reviewers collected data from each report, whether they worked independently, any processes for obtaining or confirming data from study investigators, and if applicable, details of automation tools used in the process.	Page 4
Data items	10a	List and define all outcomes for which data were sought. Specify whether all results that were compatible with each outcome domain in each study were sought (e.g. for all measures, time points, analyses), and if not, the methods used to decide which results to collect.	Page 4 and Supplementary Table 2
	10b	List and define all other variables for which data were sought (e.g. participant and intervention characteristics, funding sources). Describe any assumptions made about any missing or unclear information.	Page 4
Study risk of bias assessment	11	Specify the methods used to assess risk of bias in the included studies, including details of the tool(s) used, how many reviewers assessed each study and whether they worked independently, and if applicable, details of automation tools used in the process.	Page 4
Effect measures	12	Specify for each outcome the effect measure(s) (e.g. risk ratio, mean difference) used in the synthesis or presentation of results.	Page 4 and Supplementary Table 2
Synthesis	13a	Describe the processes used to decide which studies were eligible for each synthesis (e.g. tabulating the study	Page 4 and

Section and Topic	Item #	Checklist item	Location where item is reported
methods		intervention characteristics and comparing against the planned groups for each synthesis (item #5)).	Supplementary Table 1 and 2
	13b	Describe any methods required to prepare the data for presentation or synthesis, such as handling of missing summary statistics, or data conversions.	Page 3-4
	13c	Describe any methods used to tabulate or visually display results of individual studies and syntheses.	Page 3-4
	13d	Describe any methods used to synthesize results and provide a rationale for the choice(s). If meta-analysis was performed, describe the model(s), method(s) to identify the presence and extent of statistical heterogeneity, and software package(s) used.	Page 3-4
	13e	Describe any methods used to explore possible causes of heterogeneity among study results (e.g. subgroup analysis, meta-regression).	N/A
	13f	Describe any sensitivity analyses conducted to assess robustness of the synthesized results.	N/A
Reporting bias assessment	14	Describe any methods used to assess risk of bias due to missing results in a synthesis (arising from reporting biases).	Pages 4, 8 Supplementary Table 3
Certainty assessment	15	Describe any methods used to assess certainty (or confidence) in the body of evidence for an outcome.	N/A
**RESULTS**			
Study selection	16a	Describe the results of the search and selection process, from the number of records identified in the search to the number of studies included in the review, ideally using a flow diagram.	Page 4, Table 1 and Supplementary Figure 1
	16b	Cite studies that might appear to meet the inclusion criteria, but which were excluded, and explain why they were excluded.	Page 4 and Supplementary Figure 1
Study characteristics	17	Cite each included study and present its characteristics.	Page 5-8 and Table 1 and Supplementary Figure 1
Risk of bias in studies	18	Present assessments of risk of bias for each included study.	Page 8 and Supplementary Table 3
Results of individual studies	19	For all outcomes, present, for each study: (a) summary statistics for each group (where appropriate) and (b) an effect estimate and its precision (e.g. confidence/credible interval), ideally using structured tables or plots.	Tables 1-3
Results of	20a	For each synthesis, briefly summarise the characteristics and risk of bias among contributing studies.	Page 4-9 and Table 1-2

Section and Topic	Item #	Checklist item	Location where item is reported
syntheses			Supplementary Table 3
	20b	Present results of all statistical syntheses conducted. If meta-analysis was done, present for each the summary estimate and its precision (e.g. confidence/credible interval) and measures of statistical heterogeneity. If comparing groups, describe the direction of the effect.	N/A
	20c	Present results of all investigations of possible causes of heterogeneity among study results.	N/A
	20d	Present results of all sensitivity analyses conducted to assess the robustness of the synthesized results.	N/A
Reporting biases	21	Present assessments of risk of bias due to missing results (arising from reporting biases) for each synthesis assessed.	N/A
Certainty of evidence	22	Present assessments of certainty (or confidence) in the body of evidence for each outcome assessed.	N/A
**DISCUSSION**			
Discussion	23a	Provide a general interpretation of the results in the context of other evidence.	Pages 9-14
	23b	Discuss any limitations of the evidence included in the review.	Page 14
	23c	Discuss any limitations of the review processes used.	Page 14
	23d	Discuss implications of the results for practice, policy, and future research.	Pages 13-14
**OTHER INFORMATION**			
Registration and protocol	24a	Provide registration information for the review, including register name and registration number, or state that the review was not registered.	Not registered
	24b	Indicate where the review protocol can be accessed, or state that a protocol was not prepared.	N/A
	24c	Describe and explain any amendments to information provided at registration or in the protocol.	N/A
Support	25	Describe sources of financial or non-financial support for the review, and the role of the funders or sponsors in the review.	Page 15
Competing interests	26	Declare any competing interests of review authors.	Page 15
Availability of data, code and other materials	27	Report which of the following are publicly available and where they can be found: template data collection forms; data extracted from included studies; data used for all analyses; analytic code; any other materials used in the review.	Page 15
